# Evaluation of antibacterial and antifungal activity of ethanolic extract of *Cochlospermum regium *(Cochlospermaceae) leaf, a medicinal plant from the Cerrado of Brazil

**DOI:** 10.1186/1753-6561-8-S4-P72

**Published:** 2014-10-01

**Authors:** José Lourenço dos Santos Cunha E Silva, Bruna de Paula Bicudo, Allan Belarmino Rodrigues, Milena Mariano Mendonça, Rodrigo Raghiant Borges, Adriana Araújo de Almeida, Kelly Mari Pires de Oliveira

**Affiliations:** 1 Faculty of Biological and Environmental Sciences, Federal University of Grande Dourados, Unit II: Highway Dourados - Itahum, Km 12; 79.804-970; Dourados, MS, Brazil; 2 Faculty of Health Science, Federal University of Grande Dourados, Unit II: Highway Dourados - Itahum, Km 12; 79.804-970; Dourados, MS, Brazil

## Background

Bioprospecting of medicinal plants is a promising activity on biotechnology researches and detection of new compounds with biological activities of interest in health. Studies on medicinal plants of the brazilian Cerrado, still limited across the wide biodiversity found in this biome, it is an important source for the treatment several diseases, including fungal and bacterial infections [5]. *Cochlospermum regium *(Cochlospermaceae), popularly known as "algodãozinho do cerrado" or "algodãozinho-do-campo", is a plant used in brazilian folk medicine. Among the various medicinal properties attributed to the plant stands out its use to treat diseases of infectious etiology as well as inflammation [3]. The purpose of this study was to evaluate the antibacterial and antifungal activity of the leaf extract *Cochlospermum regium *against Gram-positive *Staphylococcus aureus *ATCC 29213, *Enterococcus faecalis *ATCC 51299, Gram-negative bacteria *Escherichia coli *ATCC 25922 and *Pseudomonas aeruginosa *ATCC 27853, and against the yeast *Candida albicans *ATCC 90028, *Candida tropicalis *ATCC 750, *Candida glabrata *ATCC 2001, *Candida krusei *ATCC 6558.

## Methods

To microbiological evaluation, the techniques used were Minimum Inhibitory Concentration (MIC), Minimum Fungicidal Concentration (MFC) and Minimum Bactericidal Concentration (MBC). The leaves were collected at farm Santa Madalena (S 22 ° 08 '18.3'' / W 055 ° 08 '23. 7'') in December 2012. Then were evaporated in rota-evaporator and lyophilized preparation of the ethanol extract. The minimum inhibitory concentration (MIC) of the extract was determined by broth microdilution according to the recommendations of CLSI (Clinical and Laboratory Standards Institute) [2]. For evaluation of the fungicide was used Sabouraud Dextrose Agar (Difco) and bactericidal Mueller Hinton Agar (Difco). It is considered that the activity of the extracts tested in MIC is less than 100 μg/mL, antimicrobial activity will be assessed good; if for the antimicrobial activity of 100 to 500 μg/mL is considered medium; from 500 to 1000 μg/mL the antimicrobial activity is considered weak; and over 1000 μg/mL the extract is considered inactive [1].

## Results and conclusions

Based on these criteria, the ethanol extract of leaf showed good activity against the *C. krusei *(64 μg/mL), *Enterococcus faecalis *(4 μg/mL), *Escherichia coli *(8 μg/mL) and an acceptable effect to *Staphylococcus aureus *(512 μg/mL) and inactivates action for *C. glabrata *(1024 μg/mL). But no effect was opposite *C. albicans*, *C. tropicalis *and *Pseudomonas aeruginosa. C. krusei *is a yeast with great medical relevance and cause of candidemia in adults, especially in hospitalized patients / debilitated. The results are interesting as they show that *C. krusei *shows a greater intrinsic resistance to the drug fluconazole, which is considered a standard medication and one of the most prescribed antimicrobial [4], and showed a reasonable resultof the antimicrobial potential against the bacteria *Enterococcus faecalis *and *Escherichia coli *may be an alternative for treatment of infections caused by them. This study confirms the antimicrobial activity of *Cochlospermum regium *and indicates that it can be a potential candidate for the development of new therapies for the treatment of infections.
